# Supplementary material for: Templated dewetting of single-crystal sub-millimeter-long nanowires and on-chip silicon circuits
Source: Nat Commun. 2019 Dec 10;10:5632. doi: 10.1038/s41467-019-13371-3 (PMC6904683; doi:10.1038/s41467-019-13371-3)
Supplement: Supplementary file 1 — Supplementary Information [file 41467_2019_13371_MOESM1_ESM.pdf]

Supplementary Information
Templated dewetting of single-crystal, ultra-long nano-wires and on-chip silicon circuits

Monica Bollani,[1,*] Marco Salvalaglio,[2,†] Abdennacer Benali,[3] Mohammed Bouabdellaoui,[3,4] Meher Naffouti,[3,5] Mario Lodari,[1] Stefano Di Corato,[1] Alexey Fedorov,[1] Axel Voigt,[2,6] Ibtissem Fraj,[3,5] Luc Favre,[3] Jean Benoit Claude,[3] David Grosso,[3] Giuseppe Nicotra,[7] Antonio Mio,[7] Antoine Ronda,[3] Isabelle Berbezier,[3] and Marco Abbarchi[3,‡]

[1]*Istituto di Fotonica e Nanotecnologie-Consiglio Nazionale delle Ricerche, Laboratory for Nanostructure Epitaxy and Spintronics on Silicon, LNESS, Via Anzani 42, 22100 Como, Italy.*
[2]*Institute of Scientific Computing, Technische Universität Dresden, 01062 Dresden, Germany*
[3]*Aix Marseille Univ, Université de Toulon, CNRS, IM2NP, Marseille, France*
[4]*Laboratory of Physics of Condensed Matter and Renewable Energy, Faculty of Sciences and Technology, Hassan II University of Casablanca, 146 Mohammedia, Morocco*
[5]*Laboratoire de Micro-Optoélectronique et Nanostructures, Faculté des Sciences de Monastir Université de Monastir, 5019 Monastir, Tunisia*
[6]*Dresden Center for Computational Materials Science, Technische Universität Dresden, 01062 Dresden, Germany.*
[7]*CNR-IMM, Zona Industriale Strada VIII, 5, 95121 Catania, Italy*
(Dated: October 25, 2019)

SUPPLEMENTARY NOTE 1: PHASE-FIELD MODEL AND SIMULATIONS

Phase field simulations were performed exploiting the model reported in Refs. 18 and 19. A diffused domain approach is considered by accounting for the dynamics of an order parameter, $\varphi(\mathbf{x})$, with $\mathbf{x} \in \Omega$ and Ω the entire simulation domain. It is set to be $\varphi = 1$ in the solid and $\varphi = 0$ with a continuous variation in between described by

$$\varphi = \frac{1}{2}\left[1 - \tanh\left(\frac{3d(\mathbf{x})}{\epsilon}\right)\right]. \tag{1}$$

ϵ is the interface thickness between phases while $d(\mathbf{x})$ is the signed distance from the surface of the solid phase. The latter is identified as the $\varphi = 0.5$ isosurface. The model is based on the definition of an energy functional

$$\begin{aligned} F[\varphi] = &\int_\Omega \gamma(\hat{\mathbf{n}})\left(\frac{\epsilon}{2}|\nabla\varphi|^2 + \frac{1}{\epsilon}B(\varphi)\right)d\mathbf{x} + \\ &+ \int_\Omega \frac{\beta}{2\epsilon}\left(-\epsilon\nabla^2\varphi + \frac{1}{\epsilon}B'(\varphi)\right)d\mathbf{x}, \end{aligned} \tag{2}$$

with $B(\varphi) = 18\varphi^2(1-\varphi)^2$. $\gamma(\hat{\mathbf{n}})$ is the surface energy density with $\hat{\mathbf{n}} = -\nabla\varphi/|\nabla\varphi|$ the outward normal of the surface of the solid phase. The first integral of Eq. (2) accounts for the surface energy within the diffused approach, while the second one is the Willmore regularization, considered in order to tackle strong anisotropy regimes by producing rounding of the corners of faceted shapes over a region whose radius is scaling as $\sqrt{\beta}$[18,19]. The evolution law for φ reads

$$\frac{\partial\varphi}{\partial t} = M(\varphi)\nabla^2\frac{\delta F}{\delta\varphi}, \tag{3}$$

with $M(\varphi) = (2M_0/\epsilon)B(\varphi)$, M_0 a diffusivity constant and $\delta F/\delta\varphi$ the functional derivative of F with respect to φ. These equations are solved by using the Finite Element toolbox AMDiS[20,21], exploiting adaptive timestep and spatial discretization. More details about the implementation and numerics can be found in Refs. 19, 22, and 23.

Following Refs. 19, 24, and 25 we define the surface energy density as

$$\gamma(\hat{\mathbf{n}}) = \gamma_0\left[1 - \sum_i^N \alpha_i\left(\hat{\mathbf{n}}\cdot\hat{\mathbf{m}}_i\right)^{w_i}\Theta\left(\hat{\mathbf{n}}\cdot\hat{\mathbf{m}}_i\right)\right], \tag{4}$$

with $\mathbf{m}_i$ the preferential orientation, i.e. the orientations along which the surface-energy density has minima, α_i controlling the depth of energy minima, w_i controlling the range of orientations affected by a specific energy minimum and N the number of different minima. As in Ref. 25 we set the coefficient α_i in order to reproduce the main facets of Si crystals. In particular, we set

$$\alpha_i = 1 - \left(\frac{\gamma_i}{\gamma_{\langle 001\rangle}}\right)\left(1 - \alpha_{\langle 001\rangle}\right), \tag{5}$$

with $\alpha_{\langle 001\rangle}$ a reference parameter. γ_i corresponding to the preferential orientations, namely $\langle 113\rangle$, $\langle 111\rangle$, and $\langle 110\rangle$ and $\langle 001\rangle$, are taken from Ref. 26. For isotropic evolution $\gamma(\hat{\mathbf{n}}) = \gamma_0$, and γ_0 would only affect the timescale of the process. The isotropic simulation reported in the main text are obtained by setting γ_0 as the average of the γ_i values entering Eq. (5). For anisotropic surface energies the differences between γ_0 and $\gamma_0\alpha_{\langle 001\rangle}$ directly affects the strength of the anisotropy. However, from a physical point of view, γ_0 would correspond to the maximum value of the surface-energy density that it is typically unknown or difficult to be determined. In order to reproduce a faceted profile for Si we considered $\gamma_{\langle 001\rangle} \sim 8.7$ eV/nm^2 as in Ref. 26. Then we arbitrarily set $\alpha_{\langle 001\rangle} = 0.05$ and, in turn, $\gamma_0 = 9.15$eV/nm^2 because when $\hat{\mathbf{n}} = [001]$ we have $\gamma(\hat{\mathbf{n}}) = \gamma_{\langle 001\rangle} = \gamma_0(1 - \alpha_{\langle 001\rangle})$. Therefore, $\alpha_{\langle 001\rangle}$ is a parameter controlling the anisotropy degree and it has been validated a posteriori by the comparison with experiments. However, it is worth mentioning that higher degree of anisotropy would lead to more expensive simulations and exploring different degrees of anisotropy was far from the scope of the present work. The Willmore regularization parameter β is here set to 0.005. Timescale of the simulations in the main text is arbitrary.

SUPPLEMENTARY NOTE 2: SPONTANEOUS DEWETTING

Spontaneous solid state dewetting of a 12 nm thick UT-SOI can be triggered by impurities on the sample surface (Supplementary Figure 1) or from defects in the BOX as shown in reference 27. Another source of disorder found on all the samples[22] are small tips on the BOX surface and at the islands edges (respectively white and yellow arrows in Supplementary Figure 1 e)) where the surface is slightly deformed with respect to the conventional facets.

Orientation dependent dewetting: complement to the cases shown in Fig. 4 in the main text

It has been shown that is possible to control the dewetting dynamics of UT-SOI in order to form ordered arrays of isolated islands and complex nano-architectures by creating *ad hoc* dewetting fronts prior to annealing.[22,28–32] However, due to the intrinsic anisotropy and stiffness of silicon, a well-controlled outcome can be only obtained for relatively small patches, extending over a few micrometer length (patch height over lateral width, h/w $\sim$1/400) and strictly oriented along the stable dewetting fronts (the [110] and [1-10] in-plane crystallographic directions). In fact, when cutting the UT-SOI in other directions (e.g. the [010], S.I.) the film rapidly evolves in finger-like projections and then in isolated islands, as for the spontaneous dewetting case. This is illustrated in Supplementary Figure 2 where a drastic change in the number of islands per patch is found when moving from the stable front (0 degrees), to the unstable one (45 degrees).

However, for lower dewetting temperature and annealing time, it is possible to avoid the onset of the Plateau-Rayleigh instability[33,34] thus obtaining long and connected wires. Examples of split and inter-connected wires forming a complex circuit are shown in the Supplementary Figure 3 that complement the case shown in the main text Fig. 4.

SUPPLEMENTARY REFERENCES

* Corresponding author: Monica Bollani (monica.bollani@ifn.cnr.it)

† Corresponding author: Marco Salvalaglio (marco.salvalaglio@tu-dresden.de)

‡ Corresponding author: Marco Abbarchi (marco.abbarchi@im2np.fr)

1 S Torabi, J Lowengrub, A Voigt, and S Wise. A new phase-field model for strongly anisotropic systems. *P. Roy. Soc. A Math. Phys.* **465**, 1337–1359 (2009).

2 Marco Salvalaglio, Rainer Backofen, Roberto Bergamaschini, Francesco Montalenti, and Axel Voigt. Faceting of equilibrium and metastable nanostructures: a phase-field model of surface diffusion tackling realistic shapes. *Cryst Growth Des.* **15**, 2787–2794 (2015).

3 Simon Vey and Axel Voigt. Amdis: adaptive multidimensional simulations. *Comput. Vis. Sci.* **10**, 57–67 (2007).

4 T. Witkowski, S. Ling, S. Praetorius, and A. Voigt. Software concepts and numerical algorithms for a scalable adaptive parallel finite element method. *Adv. Comput. Math.* **41**, 1145 (2015).

5 Meher Naffouti et al. Complex dewetting scenarios of ultrathin silicon films for large-scale nanoarchitectures. *Sci. Adv.* **3**, eaao1472 (2017).

6 Rainer Backofen, Steven Wise, Marco Salvalaglio, and Axel Voigt. Convexity splitting in a phase field model for surface diffusion. *Int. J. Numer. Anal. Model.* **16**, 192 (2019).

7 Marco Salvalaglio, et al. Phase-field simulations of faceted Ge/Si-crystal arrays, merging into a suspended film. *Appl. Surf. Sci.* **391**, 33–38 (2017).

8 Marco Salvalaglio, Rainer Backofen, Axel Voigt, and Francesco Montalenti. Morphological Evolution of Pit-Patterned Si(001) Substrates Driven by Surface-Energy Reduction. *Nanoscale Res. Lett.* **12**, 554 (2017).

9 J.M. Bermond, J.J. Métois, X. Egéa, and F. Floret. The equilibrium shape of silicon. *Surf. Sci.* **330**, 48–60 (1995).

10 Meher Naffouti et al. Fabrication of core–shell nanostructures via silicon on insulator dewetting and germanium condensation: towards a strain tuning method for sige-based heterostructures in a three-dimensional geometry. *Nanotechnology* **27**, 305602 (2016).

11 Marco Abbarchi, et al. Wafer scale formation of monocrystalline silicon-based mie resonators via silicon-on-insulator dewetting. *ACS Nano* **8**, 11181–11190 (2014).

12 Meher Naffouti, et al. Fabrication of poly-crystalline si-based mie resonators via amorphous si on sio 2 dewetting. *Nanoscale* **8**, 7768 (2016).

13 Thomas Wood, et al. All-dielectric color filters using sige-based mie resonator arrays. *ACS Photonics* **4**, 873–883 (2017).

14 Marco Abbarchi, et al. Solid-state dewetting of single-crystal silicon on insulator: effect of annealing temperature and patch size. *Microelectron. Eng.* **190**, 1–6 (2018).

15 M Aouassa, et al. Design of free patterns of nanocrystals with ad hoc features via templated dewetting. *Appl. Phys. Lett.* **101**, 013117 (2012).

16 Robert W Day, et al. Plateau–rayleigh crystal growth of periodic shells on one-dimensional substrates. *Nat. Nanotechnol.* **10**, 345 (2015).

17 Zhaoguo Xue, et al. Engineering island-chain silicon nanowires via a droplet mediated plateau-rayleigh transformation. *Nat. Commun.* **7**, 12836 (2016).

18 S Torabi, J Lowengrub, A Voigt, and S Wise. A new phase-field model for strongly anisotropic systems. *Proceedings of the Royal Society A: Mathematical, Physical and Engineering Sciences*, 465(2105):1337–1359, 2009.

19 Marco Salvalaglio, Rainer Backofen, Roberto Bergamaschini, Francesco Montalenti, and Axel Voigt. Faceting of equilibrium and metastable nanostructures: a phase-field model of surface diffusion tackling realistic shapes. *Crystal Growth & Design*, 15(6):2787–2794, 2015.

20 Simon Vey and Axel Voigt. Amdis: adaptive multidimensional simulations. *Computing and Visualization in Science*, 10(1):57–67, 2007.

21 T. Witkowski, S. Ling, S. Praetorius, and A. Voigt. Software concepts and numerical algorithms for a scalable adaptive parallel finite element method. *Adv. Comput. Math.*, 41:1145, 2015.

22 Meher Naffouti, Rainer Backofen, Marco Salvalaglio, Thomas Bottein, Mario Lodari, Axel Voigt, Thomas David, Abdelmalek Benkouider, Ibtissem Fraj, Luc Favre, et al. Complex dewetting scenarios of ultrathin silicon films for large-scale nanoarchitectures. *Science advances*, 3(11):eaao1472, 2017.

23 Rainer Backofen, Steven Wise, Marco Salvalaglio, and Axel Voigt. Convexity splitting in a phase field model for surface diffusion. *arXiv preprint arXiv:1710.09675*, 2017.

[24] Marco Salvalaglio, Roberto Bergamaschini, Rainer Backofen, Axel Voigt, Francesco Montalenti, and Leo Miglio. Phase-field simulations of faceted Ge/Si-crystal arrays, merging into a suspended film. *Applied Surface Science*, 391:33–38, 2017.

[25] Marco Salvalaglio, Rainer Backofen, Axel Voigt, and Francesco Montalenti. Morphological Evolution of Pit-Patterned Si(001) Substrates Driven by Surface-Energy Reduction. *Nanoscale Research Letters*, 12:554, 2017.

[26] J.M. Bermond, J.J. Métois, X. Egéa, and F. Floret. The equilibrium shape of silicon. *Surface Science*, 330:48–60, 1995.

[27] Meher Naffouti, Thomas David, Abdelmalek Benkouider, Luc Favre, Martiane Cabie, Antoine Ronda, Isabelle Berbezier, and Marco Abbarchi. Fabrication of core–shell nanostructures via silicon on insulator dewetting and germanium condensation: towards a strain tuning method for sige-based heterostructures in a three-dimensional geometry. *Nanotechnology*, 27(30):305602, 2016.

[28] Marco Abbarchi, Meher Naffouti, Benjamin Vial, Abdelmalek Benkouider, Laurent Lermusiaux, Luc Favre, Antoine Ronda, Sbastien Bidault, Isabelle Berbezier, and Nicolas Bonod. Wafer scale formation of monocrystalline silicon-based mie resonators via silicon-on-insulator dewetting. *ACS Nano*, 8(11):11181–11190, 2014.

[29] Meher Naffouti, Thomas David, Abdelmalek Benkouider, Luc Favre, Antoine Ronda, Isabelle Berbezier, Sebastien Bidault, Nicolas Bonod, and Marco Abbarchi. Fabrication of polycrystalline si-based mie resonators via amorphous si on sio 2 dewetting. *Nanoscale*, 8(7):7768, 2016.

[30] Thomas Wood, Meher Naffouti, Johann Berthelot, Thomas David, Jean-Benoît Claude, Léo Métayer, Anne Delobbe, Luc Favre, Antoine Ronda, Isabelle Berbezier, et al. All-dielectric color filters using sige-based mie resonator arrays. *ACS photonics*, 4(4):873–883, 2017.

[31] Marco Abbarchi, Meher Naffouti, Mario Lodari, Marco Salvalaglio, Rainer Backofen, Thomas Bottein, Axel Voigt, Thomas David, Jean-Benoît Claude, Mohammed Bouabdellaoui, et al. Solid-state dewetting of single-crystal silicon on insulator: effect of annealing temperature and patch size. *Microelectronic Engineering*, 190:1–6, 2018.

[32] M Aouassa, I Berbezier, L Favre, A Ronda, M Bollani, R Sordan, A Delobbe, and P Sudraud. Design of free patterns of nanocrystals with ad hoc features via templated dewetting. *Applied Physics Letters*, 101(1):013117, 2012.

[33] Robert W Day, Max N Mankin, Ruixuan Gao, You-Shin No, Sun-Kyung Kim, David C Bell, Hong-Gyu Park, and Charles M Lieber. Plateau–rayleigh crystal growth of periodic shells on one-dimensional substrates. *Nature nanotechnology*, 10(4):345, 2015.

[34] Zhaoguo Xue, Mingkun Xu, Yaolong Zhao, Jimmy Wang, Xiaofan Jiang, Linwei Yu, Junzhuan Wang, Jun Xu, Yi Shi, Kunji Chen, et al. Engineering island-chain silicon nanowires via a droplet mediated plateau-rayleigh transformation. *Nature Communications*, 7:12836, 2016.

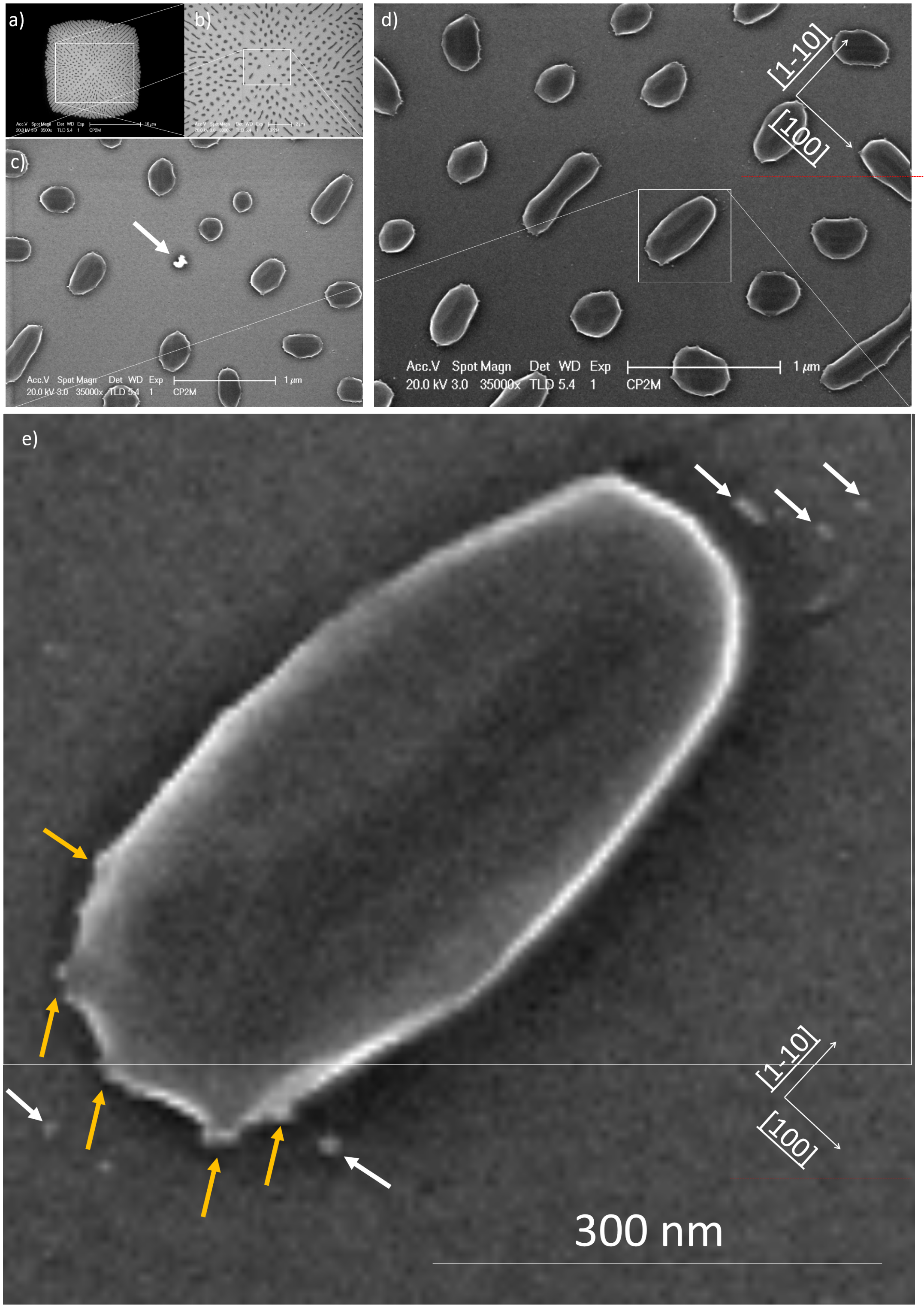

Supplementary Figure 1. SEM images of a spontaneously dewetted sample (see details in reference 27). a) Large view of a dewetted area in a sample dewetted at 780°C for 120 minutes. b) Blow up of the central area of a). c) Blow up of the central area of b). The white arrow highlight the point where the dewetting started, in presence of an impurity. More details about the starting points of spontaneous dewetting are provided in reference 27. d) High-resolution SEM of the the same sample shown in a). e) Blow up of one island from d). The white arrows highlight small tips on the denuded BOX, whereas the yellow ones highlight those in contact with the edge of the islands.

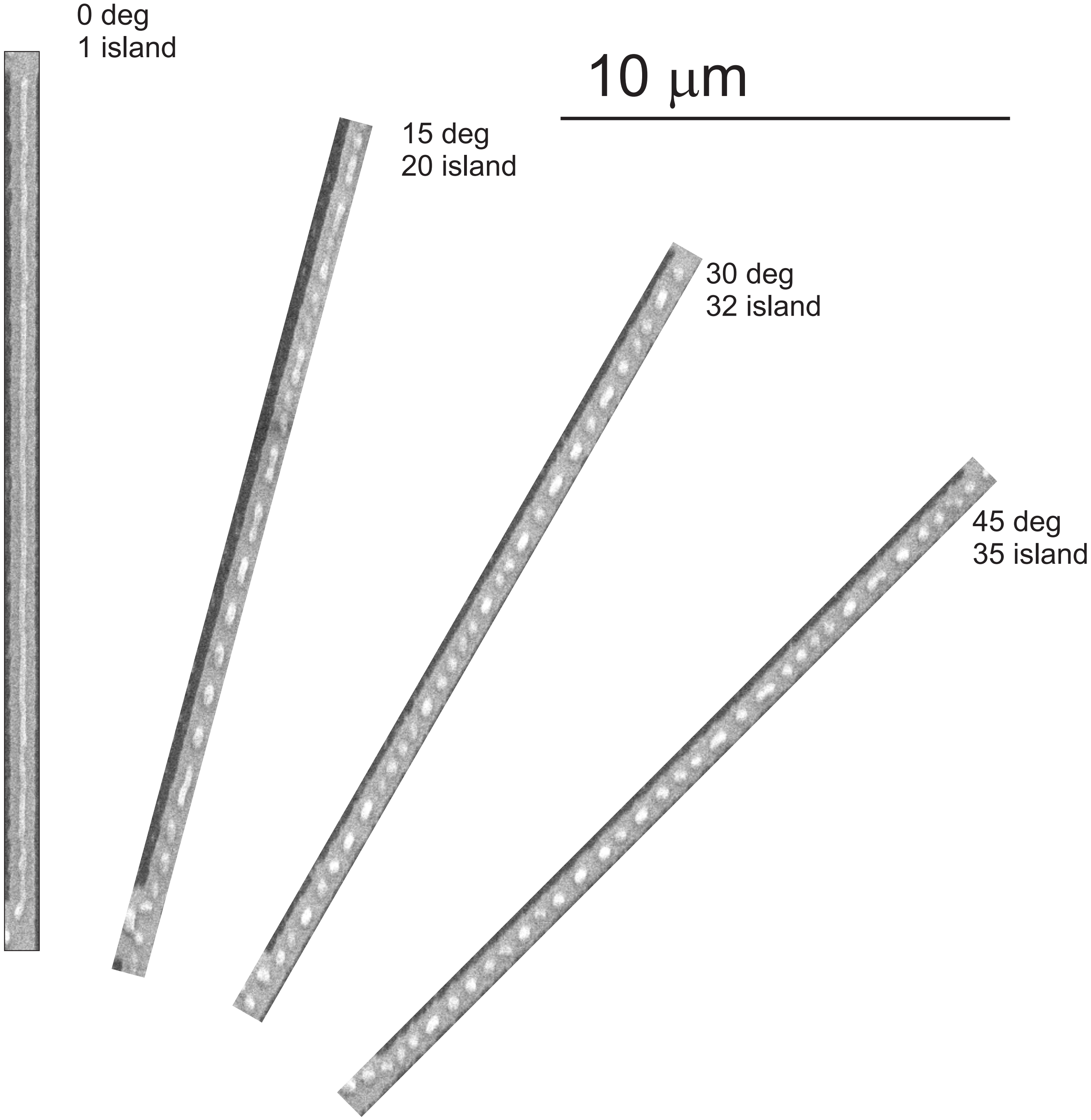

Supplementary Figure 2. SEM image of a 20 μm long patches oriented along different directions with respect to the stable [110] dewetting front taken as reference (0 degrees).

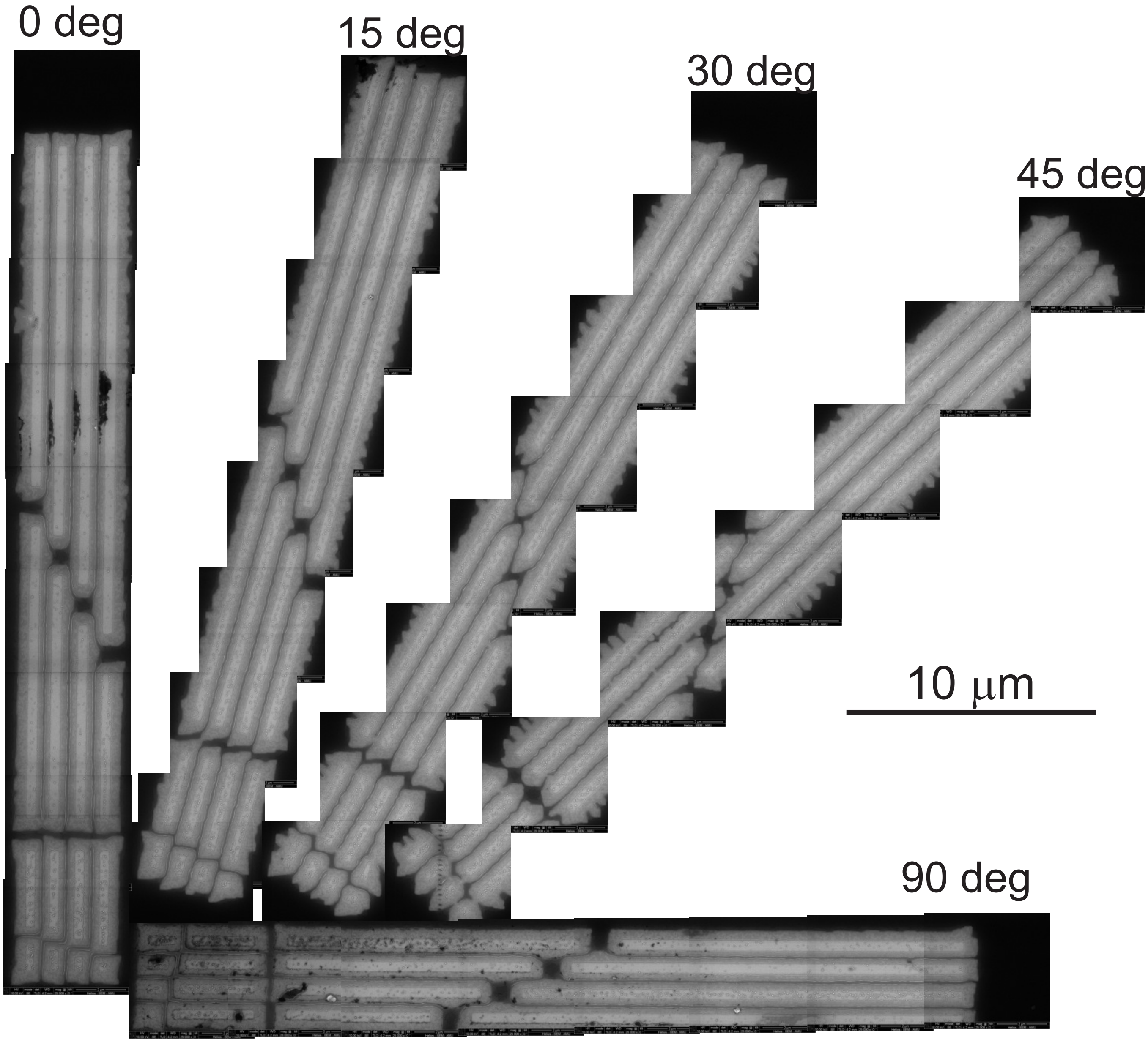

Supplementary Figure 3. Complement to the cases shown in Fig. 4 in the main text. Arrays composed of 3 wires interconnected and split etched at different orientations (changed in 15 degrees steps) with respect to the [110] direction. In this case the dewetting was induced annealing at 740 °C for 15 minutes.[22]
