# Supplementary material for: Templated dewetting of single-crystal sub-millimeter-long nanowires and on-chip silicon circuits
Source: Nat Commun. 2019 Dec 10;10:5632. doi: 10.1038/s41467-019-13371-3 (PMC6904683; doi:10.1038/s41467-019-13371-3)
Supplement: Supplementary file 3 — Description of Additional Supplementary Files [file 41467_2019_13371_MOESM3_ESM.pdf]

Description of Additional Supplementary Files

File Name: Supplementary Movie 1
Description: Full dewetting dynamics for Si wires taking into account the crystal anisotropy (faceting) corresponding to the case shown in Figure 3a in the main text (left panel)

File Name: Supplementary Movie 2
Description: Full dewetting dynamics for Si wires without taking into account the crystal anisotropy (faceting) corresponding to the case shown in Figure 3a in the main text (right panel)

File Name: Supplementary Movie 3
Description: Full dewetting dynamics for Si wires taking into account the crystal anisotropy (faceting) corresponding to the case shown in Figure 3b in the main text (left panel)

File Name: Supplementary Movie 4
Description: Full dewetting dynamics for Si wires without taking into account the crystal anisotropy (faceting) corresponding to the case shown in Figure 3b in the main text (right panel)

File Name: Supplementary Movie 5
Description: Full dewetting dynamics for Si wires taking into account the crystal anisotropy (faceting) corresponding to the case shown in Figure 3c in the main text (left panel)

File Name: Supplementary Movie 6
Description: Full dewetting dynamics for Si wires without taking into account the crystal anisotropy (faceting) corresponding to the case shown in Figure 3c in the main text (right panel)

File Name: Supplementary Data 1
Description: is a patchwork of SEM images showing the Si nanowires in their full length of 0.75 mm and it demonstrates that there are no defects nor fractures along the wires length.

File Name: Supplementary Data 2
Description: is a patchwork of SEM images showing how the Si wires break in an increasing number of islands when changing the orientation of the patches from 1 to 45 degrees with respect to a stable dewetting front.

File Name: Supplementary Data 3
Description: is a patchwork of SEM images showing Si-based complex and connected circuits oriented at 0 degrees with respect to a stable dewetting front.

File Name: Supplementary Data 4
Description: is a patchwork of SEM images showing Si-based complex and connected circuits oriented at 15 degrees with respect to a stable dewetting front.

File Name: Supplementary Data 5
Description: is a patchwork of SEM images showing Si-based complex and connected circuits oriented at 30 degrees with respect to a stable dewetting front.

File Name: Supplementary Data 6

Description: is a patchwork of SEM images showing Si-based complex and connected circuits oriented at 45 degrees with respect to a stable dewetting front.

File Name: Supplementary Data 7

Description: is a patchwork of SEM images showing Si-based complex and connected circuits oriented at 90 degrees with respect to a stable dewetting front.
